# The Effects of Different Invitation Schemes on the Use of Fecal Occult Blood Tests for Colorectal Cancer Screening: Systematic Review of Randomized Controlled Trials

**DOI:** 10.3390/cancers13071520

**Published:** 2021-03-25

**Authors:** Laura F. Gruner, Efrat L. Amitay, Thomas Heisser, Feng Guo, Tobias Niedermaier, Anton Gies, Michael Hoffmeister, Hermann Brenner

**Affiliations:** 1 German Cancer Research Center (DKFZ), Division of Clinical Epidemiology and Aging Research, 69120 Heidelberg, Germany; l.gruner@dkfz.de (L.F.G.); e.amitay@dkfz-heidelberg.de (E.L.A.); t.heisser@dkfz-heidelberg.de (T.H.); f.guo@dkfz-heidelberg.de (F.G.); t.niedermaier@dkfz-heidelberg.de (T.N.); m.hoffmeister@dkfz-heidelberg.de (M.H.); 2 Medical Faculty Heidelberg, Heidelberg University, 69120 Heidelberg, Germany; 3 Division of Preventive Oncology, German Cancer Research Center (DKFZ) and National Center for Tumor Diseases (NCT), 69120 Heidelberg, Germany; anton.gies@nct-heidelberg.de; 4 German Cancer Consortium (DKTK), German Cancer Research Center (DKFZ), 69120 Heidelberg, Germany

**Keywords:** colorectal cancer, early detection, screening, fecal occult blood tests, invitation

## Abstract

**Simple Summary:**

There is large heterogeneity in invitation schemes and participation rates in colorectal cancer screening programs offering fecal occult blood tests (nowadays mostly fecal immunochemical tests). It is unclear what the most effective invitation strategies are for fecal occult blood tests. In this systematic review, advance notification, mailed fecal occult blood test, and reminders had major, consistent, and complementary potential to increase participation in fecal occult blood test-based colorectal cancer screening. Our findings show that the effectiveness of invitations for fecal occult blood test-based colorectal cancer screening can be substantially increased across several settings by the implementation of comprehensive invitation strategies.

**Abstract:**

Personal invitations for fecal occult blood tests (nowadays mostly fecal immunochemical tests) are increasingly used to raise their usage for colorectal cancer screening. However, there is a large heterogeneity in applied invitation schemes. We aimed to review evidence for the effectiveness of various invitation schemes. The main outcome was the fecal occult blood test usage rate. A systematic search was performed in Medline and Web of Science (up to 9 July 2020). Randomized controlled trials or cluster-randomized controlled trials were eligible, which reported on general invitations for fecal occult blood test-based colorectal cancer screening sent to the general population at average colorectal cancer risk. (PROSPERO 2020 CRD42020169409). Overall, 34 studies were included. Invitations with an attached, i.e., mailed fecal occult blood test consistently increased test usage by 4–19.7% points, compared to other methods of test provision. Likewise, the introduction of advance notification consistently led to a higher usage rate, with an increase of 3.3–10.8% points. Reminders showed positive but varying effects by method. With an increase of 8.5–15.8% points, letter or email reminders were more effective than reminders by phone call or text message (0.6–6.5% points). Inconsistent results were found for financial incentives ((−8.4)–20% points) and for added or changed invitation material ((−3.5)–11.8% points). With 3.5–24.7% points, the strongest increases in use were achieved by multifaceted invitation, implementing multiple components. Any invitation scheme was superior over no invitation. Advance notification, mailing of fecal occult blood test, and reminders were consistently shown to have major, complementary potential to increase participation in fecal occult blood test-based colorectal cancer screening settings.

## 1. Introduction

Colorectal cancer (CRC) is the third most common cancer globally, accounting for approximately 1.8 million new cancer cases and 900,000 cancer-related deaths per year [1]. Screening with fecal occult blood tests, i.e., guaiac-based fecal occult blood tests (gFOBTs) or fecal immunochemical tests (FITs), and endoscopic methods have been shown by randomized, observational, and modeling studies to effectively reduce CRC incidence and mortality [2,3,4]. For gFOBTs, randomized clinical trials showed a reduction in CRC-related mortality by up to 30% with regular annual or biennial usage [3]. Thus, many European countries have implemented fecal occult blood test-based population-wide screening programs, and the majority of them have meanwhile switched from the long-established gFOBTs to FITs, [5] which have a substantially higher sensitivity for the detection of CRC and its precursors [6,7].

Still, participation rates are often low, especially where gFOBTs/FITs are offered in opportunistic screening programs (i.e., without targeted invitations of the population entitled to screening) [5]. An increasing number of differing schemes have been introduced to invite the entitled population to gFOBT/FIT screenings in organized screening programs, using different approaches to enhance adherence, such as different methods of gFOBT/FIT provision (e.g., by mail or low-threshold pick up), advance notification, or reminder systems, among others.

In order to inform decisions regarding design elements of screening programs, we aimed to provide a systematic review of the evidence on effectiveness of various invitation measures to enhance adherence to gFOBT/FIT-based screening, from randomized and cluster-randomized controlled trials that were conducted in the general population at average CRC risk.

## 2. Methods

### 2.1. Data Sources and Searches

This systematic review was performed according to the PRISMA guidelines [8] (PROSPERO 2020 CRD42020169409).

The literature was searched systematically on January 23, 2020 (repeated on 9 July 2020) in Medline (legacy PubMed) and Web of Science using keywords and MeSH terms but no restrictions for language or date. Additionally, reference lists of included papers and of relevant previous systematic reviews, identified by the search strategy, were searched. The specific search terms are provided in the Supplementary Materials. Duplicates were removed, and titles and abstracts were screened for relevant articles. Selection of articles based on pre-defined eligibility criteria was performed by two authors (L.F.G., E.L.A.).

### 2.2. Study Selection

Eligible studies included randomized or cluster-randomized controlled trials investigating invitation schemes for gFOBT/FIT-based CRC screening in the general population with average CRC risk (without specific focus, e.g., on study volunteers or previous screening users only). Other interventions to enhance CRC screening were not included (e.g., personal navigator). The main outcome was gFOBT/FIT usage rate achieved in different invitation schemes in the general population, indicated by the difference in %-points to a reference group within a certain pre-defined time period (i.e., follow-up after invitation).

### 2.3. Data Extraction and Quality Assessment

Predefined forms were used for data extraction, which was performed independently by L.F.G. and E.L.A. Detailed data on applied invitation schemes were collected. Additionally, L.F.G. and E.L.A. individually assessed study quality (on study level) using the revised Cochrane risk-of-bias tool for randomized trials (RoB 2), which allowed the assessment of the risk of bias in five distinct domains (randomization process, deviations from intended interventions, missing outcome data, measurement of the outcome, and selection of the reported result) [9]. The extracted data were compared between authors and disagreements were solved by discussion.

### 2.4. Data Synthesis

Results of eligible studies were displayed in overview tables and figures. Meta-analyses were not performed due to the large heterogeneity between invitation schemes in the included studies.

## 3. Results

### 3.1. Study Selection

**Figure 1:**
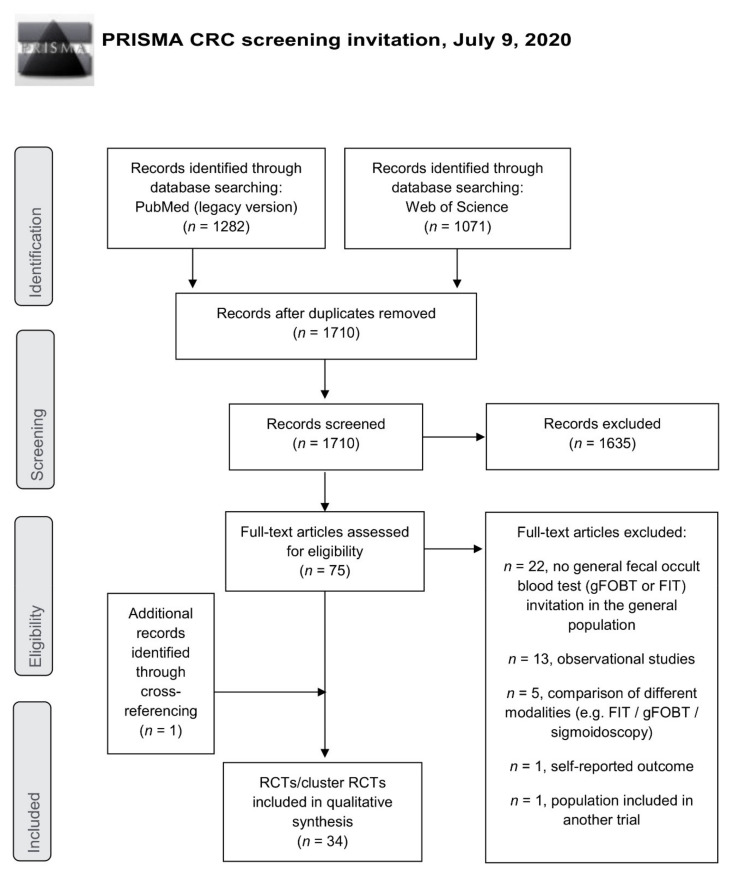
PRISMA flow diagram. CRC = colorectal cancer; FIT = fecal immunochemical test; gFOBT = guaiac-based fecal occult blood test; RCT = randomized controlled trial.

The systematic search identified 2353 records, 643 of which were duplicates (Figure 1, PRISMA flow diagram). Title and abstract screening was performed and 1635 of 1710 records were not eligible and excluded. 75 full-texts were assessed for eligibility according to pre-specified criteria: 42 were excluded (no general gFOBT/FIT invitation in the general population, *n* = 22; observational study, *n* = 13; comparison of modalities (e.g., FIT, gFOBT, or sigmoidoscopy), *n* = 5; self-reported outcome, *n* = 1; or population included in another trial, *n* = 1). One additional study was identified by cross-referencing. Thus, the systematic review comprises 34 studies [10,11,12,13,14,15,16,17,18,19,20,21,22,23,24,25,26,27,28,29,30,31,32,33,34,35,36,37,38,39,40,41,42,43].

### 3.2. Study Characteristics

The included studies were from 11 countries (14 from the USA, 6 from England, 3 from Israel, 2 from Germany, 2 from the Netherlands, 2 from Scotland, 1 from Australia, 1 from France, 1 from Italy, 1 from Portugal, and 1 from Spain), and were published between 1991 and 2020 (Table 1). Sample size ranged between 202 and 150,417, and the targeted age groups between 45 and 80 years. All reported results for one round of invitation, and follow-up of gFOBT/FIT usage ranged between 3 and 12 months. 16 studies used gFOBT, 16 used FIT, and two did not specify.

### 3.3. Quality Assessment

Quality assessment with the RoB 2 tool resulted in a low risk of bias for 14 studies [10,11,13,14,16,19,26,28,32,34,38,39,40,42], 19 studies with some concerns [12,15,17,18,20,21,22,23,24,25,27,29,30,31,33,36,37,41,43], and one [35] at high risk of bias (Table S1). Concerns were mainly due to the ambiguity of randomization and baseline characteristics (domain 1 “randomization process”), and a lack of pre-specified protocol or analysis plan (domain 5 “selection of the reported result”).

## 4. Study Settings and Populations

**Figure 2:**
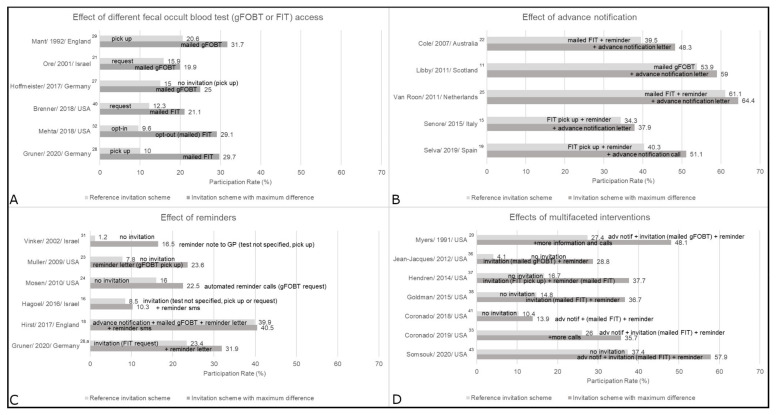
Main results by intervention approach: (**A**) fecal occult blood test (gFOBT or FIT) access, (**B**) advance notification, (**C**) reminders, and (**D**) multifaceted interventions. This figure shows the reference group and the group with the biggest difference, groups in between were dropped. ^a^ Randomized sub-study. adv notif = advance notification; FIT = fecal immunochemical test; gFOBT = guaiac-based fecal occult blood test; GP = general practitioner; sms = text message.

**Figure 3:**
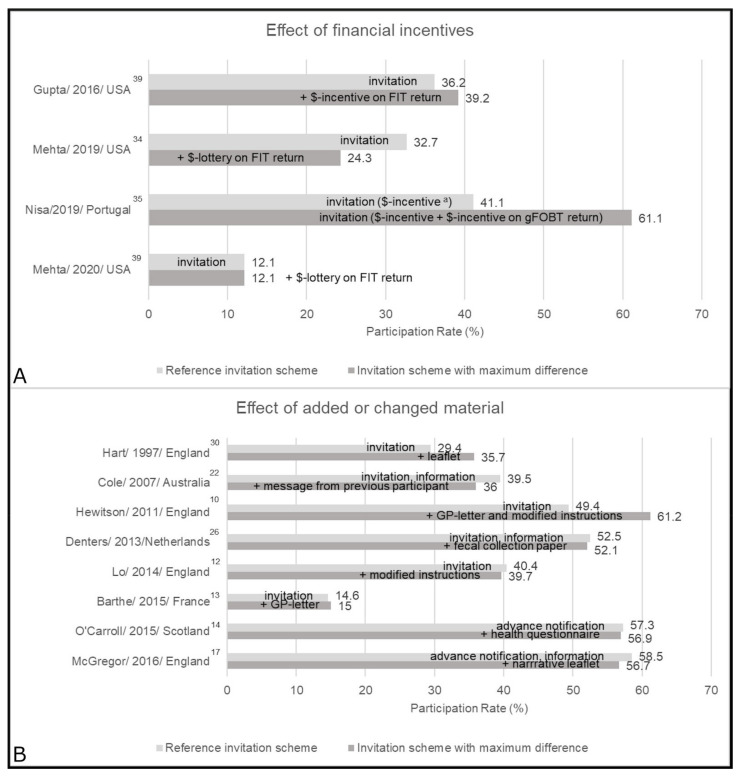
Main results by intervention approach: (**A**) financial incentives, and (**B**) added or changed material. This figure shows the reference group and the group with the biggest difference, groups in between were dropped ^a^. The sum of the divided incentive equals the once only incentive. FIT = fecal immunochemical test; gFOBT = guaiac-based fecal occult blood test; GP = general practitioner; $ = financial.

Most studies were from population-based settings (*n* = 19) (Table 1), where service organizations mainly sent the invitations. Ten were performed in national screening programs [10,11,12,13,14,15,16,17,18,19], and nine in other population-based settings (e.g., within a specific region or health maintenance organization) [20,21,22,23,24,25,26,27,28]. Primary care settings (*n* = 7) included practices [29,30], clinics [31], university clinics [32,34], or health centers [33,35], and most invitations were sent on behalf of the physicians. Eight studies (all from the USA) included predominantly low-income or uninsured individuals in safety net [37,39,43], federally qualified health centers (FQHC) [36,38,41,42], and Medicaid settings [40]. Primarily, the health system sent the invitation. In most studies, no invitation was sent to individuals with recent screening activities, e.g., gFOBT/FIT in the past year, endoscopic screening within up to 10 years [13,16,19,23,24,25,26,28,32,34,35,36,37,38,39,40,41,42,43], or (family-) history of CRC or other severe medical issues [10,15,16,19,23,24,25,28,29,32,33,34,36,37,38,39,40,41,42,43]. Main results are presented by different intervention approaches in Figure 2 and Figure 3, and by different settings in Tables S2–S5.

### 4.1. Effect of Different gFOBT/FIT Access

Provision of gFOBT/FIT can be categorized into directly mailed, sent upon request, or available for pick up, e.g., from the general practitioner (GP). Different access methods were investigated in three population-based studies [21,27,28], two from primary care [29,32], and one among Medicaid beneficiaries [40] (Figure 2; Tables S3–S5). In all respective studies, invitation schemes with mailed gFOBT/FIT consistently resulted in the highest usage. When compared to pick up options, mailed gFOBT/FIT increased usage in one study from England by 11.1% points for gFOBT [29], and in two studies from Germany by 10% points for gFOBT [27] and 19.7% points for FITs [28]. However, request options (by reply mail, fax, email, or online form) led to an almost equivalent increase of 17.7% points in the latter study [28]. While Ore et al. [21] also reported almost equivalent effects of a request card and mailed gFOBT [21], an invitation letter with the option to opt-out of mailed FIT was superior over opt-in by almost 20% points [32]. One study conducted in the US Medicaid setting [40] observed higher usage (by 8.8% points) upon reminders with mailed FIT as compared to reminders with a request option.

### 4.2. Effect of Advance Notification

Advance notification was investigated in five population-based studies [11,15,19,22,25] (Tables S2 and S3), and consistently resulted in increased usage (Figure 2). Selva et al. [19] found an increase of 10.8% points by calling participants in advance to the standard national invitation procedure. The other four studies used letters to announce the upcoming invitation and observed increases in gFOBT/FIT usage rates between 3.3% points and 8.8% points [11,15,22,25].

### 4.3. Effect of Reminders

Five population-based studies [16,18,23,24,28] and one primary care study [31] (Tables S2–S4) investigated reminders. Three did not specify any previous invitations [23,24,31]. All observed positive, albeit variable effects by method (Figure 2). In national screening programs in England and Israel, different text message reminders led to marginal increases in gFOBT/FIT usage by 0.6% points [18] and 0.7–1.8% points, respectively [16]. Automated reminder calls with a request option increased usage by 6.5% points in an American study [24]. In a randomized sub-study [28], reminder letters increased usage by 7.5–8.5% points. Muller et al. [23] used reminder letters and emails, which both were effective and increased usage of any CRC screening (including gFOBT) by 15.8% points and 14.9% points, respectively. Vinker et al. [31] provided data on a reminder note from the GP, or a letter or call addressed to the invitee, compared to usual care without invitation. The increase in usage rates was 15.3 and 10.7% points, respectively.

### 4.4. Effect of Financial Incentives

The effects of financial incentives were mixed in primary care [34,35], safety net [39], and FQHC settings [42] (Figure 3; Tables S4–S5). Three studies [34,39,42] concluded that different financial incentives (around $5–10 or chance of winning $100) did not increase FIT usage rate. In a study by Nisa et al. [35] (at high risk of bias) a divided incentive (5€ attached, and 5€ upon gFOBT usage) yielded a 20% points higher usage rate as compared to one attached incentive of 10€.

### 4.5. Effect of Added or Changed Material

The effects of added or changed material (e.g., enhanced leaflets, or GP signature) were mixed in seven population-based studies [10,12,13,14,17,22,26] and one from primary care [30] (Figure 3; Tables S2–S4). A letter from the GP did not show an effect in one study [13], but increased usage by 11.8% points in combination with enhanced instructions [10]. In 1997, Hart et al. [30] observed positive effects of an additional information leaflet, while the addition of a second leaflet [17] or modified instructions [12] resulted in minor negative changes in the more recent British national screening program. In Australia, specific messages on risk or from previous participants led to changes of 0.8% points and −3.5% points, respectively [22]. Health questionnaires [14] or provision of fecal collection paper [26] showed no effect.

### 4.6. Effects of Multifaceted Interventions

Multifaceted invitations, i.e., simultaneous implementation of several interventions, were investigated in population-based [20], primary care [33], and safety net or FQHC settings [36,37,38,41,43] (Figure 2; Tables S3–S5). Combinations of advance notification, mailed gFOBT/FIT, and reminders were investigated by two studies [20,33], with the most comprehensive approaches leading to the most pronounced increases, by 20.7% points and 9.7% points, respectively. In the safety net and FQHC studies, multifaceted invitations were compared against usual care without any invitation, and all [36,37,38,43] but one [41] observed an increase of >20% points.

### 4.7. Usage Rates without Invitation

Without invitation (i.e., opportunistic use of screening), usage rates were between 1 and 17% [23,24,27,31,36,37,38,41] (Tables S3–S5). One study [43] with higher rates used medical assistants and additional software in usual care. In the respective studies, any personal invitation yielded increased gFOBT/FIT usage rates.

## 5. Discussion

The aim of this systematic review was to summarize studies that investigated the effects of different invitation schemes for gFOBTs/FITs on their utilization in the general population. Consistently, mailed gFOBT/FIT, advance notification, and reminders increased usage rates. An increase of 4–19.7% points was achieved for mailed gFOBT/FIT, while the implementation of advance notification increased usage by 3.3–10.8% points. Reminders showed positive but varying effects by method. With an increase of 8.5–15.8% points, letter or email reminders were more effective than reminders by phone call or text message (maximum change of 0.6–6.5% points). Findings on the effects of financial incentives were inconsistent (maximum change of (−8.4)–20% points), and though results for added or changed invitation material were also mixed (maximum change of (−3.5)–11.8% points), enhanced instructions and a GP-endorsement letter increased gFOBT usage by up to 12% points. Studies in which several interventions were implemented at the same time (multifaceted invitation) all reported higher gFOBT/FIT usage rates than controls, with an increase in the range of 3.5 to 24.7% points. Any invitation scheme was superior over no invitation.

To our knowledge, this is the first systematic review of randomized and cluster-randomized trials to provide comprehensive details on individual design aspects and the effectiveness of general invitations for gFOBT/FIT addressed at the general population. It is different from five previous systematic reviews, which summarized data on how to increase gFOBT/FIT usage, but included all kinds of interventions (in addition to general invitations) and outcomes [44,45,46], had a specific focus on mailed outreach [47,48] or on FITs [44], or also reviewed non-randomized studies [44,47,48]. Although these reviews also included studies on different invitation schemes, many of these were not addressed at the general population but at specific groups, such as study volunteers or previous test users only [44,45,46,47,48], which potentially limits direct comparison and transferability to routine practice.

Previous reviews by Rat et al. [45] and by Issaka et al. [44], which described any interventions to increase usage of gFOBTs/FITs, reported positive effects for mailed gFOBTs/FITs, advance notification, and reminders. Furthermore, calls from personal advisors [45] or FITs offered in combination with vaccinations [44] were other non-invitation interventions that showed the potential to increase FOBT usage.

The majority of population-based CRC screening programs in the European Union have meanwhile implemented the concept of invitation letters with attached gFOBT/FIT, which are sent out by screening hubs [49]. Nevertheless, there are countries, such as Germany, where FITs remain to be picked up at doctors’ offices and usage rates have remained very low, which suggests room for major improvement. The second European screening report indicates that these programs are publicly funded and that health insurance plans act as funding sources in more than half of these programs [50]. At 49.5% (range: 22.8–71.3%), overall participation was higher in countries with FIT-based approaches than in those that used the gFOBT (overall usage at 33.2%, range: 4.5–66.6%) [51]. Thus, it is possible that the invitation schemes presented in this systematic review, which used gFOBT, would have achieved even higher response rates by using the FIT.

According to EU Guidelines, the desirable usage rate of CRC screening is >65% [51]. In the current review, one Dutch population-based study reported FIT usage rates close to this recommendation at 64.4% [25]. In this invitation scheme an advance notification letter with information was sent. Two weeks later, individuals received an invitation letter, which included an information brochure, an FIT, an instruction leaflet for sample taking, an informed consent form, and a postpaid reply envelope. Those who did not return the FIT within six weeks received a reminder letter [25]. A comparable approach was studied in a Dutch population-based study [52], and several rounds of biannual invitations were well accepted, with cumulative usage rates of >70% [53,54]. Additionally, the first five years of the Dutch national screening program (including advance notification one week prior to an invitation letter with an FIT (postpaid return), followed by a reminder letter) [55] also showed usage rates >70% [55,56,57,58,59].

Access to gFOBT/FIT through direct mailing was shown to be more effective than other methods for providing access within six studies investigating this aspect, and, moreover, was the most commonly used access method across all included studies. Yet, it is also likely to be the most expensive way to provide gFOBTs/FITs and to create the highest number of unused tests. To date, few trials have investigated the effect of request options for gFOBTs/FITs, which request only a small additional effort from the individual as compared to directly mailed tests, but might save economic and environmental costs. In this systematic review, promising results were found in population-based settings, where usage rates of requested gFOBTs/FITs were almost comparable to mailed tests [21,28]. The acceptance of request options for more user-friendly FITs [28] was higher than for gFOBTs [21], which is in line with previous experiences [51,60]. If, and to what extent, the provision of gFOBTs/FITs upon easy access is cost-effective should thus be addressed by further comprehensive health economical evaluations. It might additionally be relevant to offer postpaid return of the used gFOBTs/FITs to avoid barriers such as having to take the test to the GP or paying for postage. Although in this systematic review the results for GP-involvement were mixed, more than half of the studies conducted in the primary care setting already included a primary care physician or clinician in the invitation process (e.g., GP-signature by default) which might have contributed to the respective usage. Furthermore, the effectiveness of the different invitation schemes in sub-groups of the target population (such as gender, age, and socioeconomic status) was not consistently reported in the included studies, and should be investigated more in future research.

Specific strengths of this systematic review include a detailed description of gFOBT/FIT invitation schemes in the general, screening-eligible population, which were investigated in randomized or cluster-randomized controlled trials. It complements previous systematic reviews [13] with new studies on the specific effects of gFOBT/FIT provision in the frame of invitations, while studies with focus on specific groups or other interventions were excluded to enhance comparability to routine invitation practice. Nevertheless, this systematic review has several limitations. Characteristics (including settings, populations, and sample size, but also sender of the invitation, follow-up duration, and specific procedures) varied greatly among the included studies, therefore limiting direct comparability of the results. Furthermore, invitation procedures were described according to the used terminology in the respective publications, and although some studies stated to investigate a reminder [23,24,31,40], it was the first described contact and might thus be comparable to an initial invitation. Studies on multifaceted interventions were all performed in the USA, however, it can be assumed that comprehensive invitation models are effective in other countries as well, where more specific design aspects were investigated. Due to the large heterogeneity between studies, no quantitative measurement of comparison for the most effective invitation scheme was made. However, it can be assumed from the summarized data that a comprehensive invitation strategy including several contact attempts combined with low-threshold access to gFOBT/FIT (e.g., a combination of advanced notification, mailed gFOBT/FIT, and a reminder) has the strong potential to increase usage.

Some of the frequently observed risks of bias, such as missing pre-specified analysis plans, possibly did not influence the outcome gFOBT/FIT usage, which was either yes or no. Furthermore, it is likely that in most trials without informed consent, the target population did not even know that they were included in a study, and were therefore completely blinded.

In conclusion, this systematic review summarizes findings from controlled trials on CRC screening invitations, in which mailed gFOBT/FIT, advance notification, and reminders consistently show the potential for high test usage across various settings. A synergistic effect for a substantial increase in CRC screening usage might be achievable by combining various interventions. Low-barrier provision upon request appears to be an almost equally effective alternative to direct mailing of gFOBT/FIT, and could have major economical and environmental advantages in population-wide screening programs.

## Figures and Tables

**Table 1:** Characteristics of randomized and cluster-randomized studies included in systematic review.

	First Author/Year/Country	Age Range	*N* Sample (% Females)	Setting	Sender or Send on Behalf of	gFOBT or FIT (Brand)	F-Up After Invitation
	Population-Based Studies (National Screening Programs)						
**1**	Hewitson/ 2011/ England [10]	60–75	1288 (52.6%)	NSP	screening hubs	gFOBT (/)	20 wk
**2**	Libby/ 2011/ Scotland [11]	50–74	59,953 (51.3%)	NSP	bowel screening center	gFOBT (/)	26–32 wk
**3**	Lo/ 2014/ England [12],^a^	60–69	23,182 (/)	NSP	screening hubs	gFOBT (/)	/
**4**	Barthe/ 2015/ France [13],^a^	50–74	3422 (56.7%)	NSP	district screening org	gFOBT (/)	6 mo
**5**	O’Carroll/ 2015/ Scotland [14]	50–74	59,366 (51%)	NSP	mail company	gFOBT (/)	6 mo
**6**	Senore/ 2015/ Italy [15]	50–69	20,701 (53.2%)	NSP	screening org	FIT (/)	3 mo
**7**	Hagoel/ 2016/ Israel [16]	50–74	48,091 (51%)	NSP	nat prog health services	/	6 mo
**8**	McGregor/ 2016/ England [17],^a^	59–74	150,417 (51.2%)	NSP	screening hubs	gFOBT (/)	18 wk
**9**	Hirst/ 2017/ England [18]	60–74	8269 (52%)	NSP	mobile health org	gFOBT (/)	18 wk
**10**	Selva/ 2019/ Spain [19]	50–69	492 (54.7%)	NSP	regional staff	FIT (/)	6 mo
**Population-Based Studies (Other)**							
**11**	Myers/ 1991/ USA [20]	50–74	2201 (47%)	HMO	central screening office	gFOBT (HemaWipe)	90 d
**12**	Ore/ 2001/ Israel [21]	50–74	1940 (50.2%)	HMO	/	gFOBT (Haemoccult II S)	4 mo
**13**	Cole/ 2007/ Australia [22]	50–74	2400 (/)	comm prog	central screening facility	FIT (InSures)	12 wk
**14**	Muller/ 2009/ USA [23]	50–80	1397 (/)	HMO	researchers	gFOBT (/)	3 mo
**15**	Mosen/ 2010/ USA [24]	51–80	5905 (50.2%)	HMO	call scripts	gFOBT (/)	6 mo
**16**	Van Roon/ 2011/ Netherlands [25]	50–74	4784 (50.9%)	one region	/	FIT (OC Sensor)	/
**17**	Denters/ 2013/ Netherlands [26]	50–75	10,265 (51%)	pilot prog	regional center	FIT (OC Sensor)	/
**18**	Hoffmeister/ 2017/ Germany [27]	50	18,560 (/)	one region	education minister	gFOBT (/)	1 y
**19**	Gruner/ 2020/ Germany [28]	50–54	17,532 (46.3%)	HMO	HMO	FIT (OC Sensor)	1 y
**Primary Care-Based Studies**							
**20**	Mant/ 1992/ England [29]	45–64	1588 (47.9%)	PC practice	general practice	gFOBT (Haemoccult)	/
**21**	Hart/ 1997/ England [30]	61–70	1571 (52.8%)	PC practice	signed by senior partner	gFOBT (Haemoccult)	/
**22**	Vinker/ 2002/ Israel [31]	50–75	2315 (52.2%)	PC clinics	/	/	1 y
**23**	Mehta/ 2018/ USA [32]	50–74	314 (53.2%)	PC university clinic	researchers	FIT (OC Sensor)	3 mo
**24**	Coronado/ 2019/ USA [33]	50–74	1767 (56.9%)	comm health center	health center	FIT (InSure)	6 mo
**25**	Mehta/ 2019/ USA [34]	50–75	897 (56%)	PC university clinic	PC clinician	FIT (/)	6 mo
**26**	Nisa/ 2019/ Portugal [35]	50–74	1652 (51%)	public health center	head physician	gFOBT (/)	/
**Studies from Safety Net, FQHC, and Medicaid Settings**							
**27**	Jean-Jacques/ 2012/ USA [36]	50–80	202 (61.8%)	FQHC	medical professional	gFOBT (Hemoccult II S)	4 mo
**28**	Hendren/ 2014/ USA [37]	50–74	240 (/)	Safety net	outreach worker	FIT (/)	1 y
**29**	Goldman/ 2015/ USA [38]	50–74	420 (66%)	FQHC	health center network	FIT (OC Light)	6 mo
**30**	Gupta/ 2016/ USA [39]	50–64	8565 (61.8%)	Safety net	safety net health system	FIT (OC Sensor)	6 mo
**31**	Brenner/ 2018/ USA [40]	52–64	1490 (51.7%)	Medicaid	health department	FIT (OC Light)	12 mo
**32**	Coronado/ 2018/ USA [41],^a^	50–74	41,193 (55.8%)	FQHC	clinic staff	FIT (/)	12 mo
**33**	Mehta/ 2020/ USA [42]	50–74	281 (63%)	FQHC	clinic sms platform	FIT	3 mo
**34**	Somsouk/ 2020/ USA [43]	50–75	10,820 (47.1%)	Safety net	study team	FIT (OC Light / Sensor)	1 y

^a^ cluster-randomized study. comm = community; d = days; FIT = fecal immunochemical test; FQHC = federally qualified health centers; F-up = Follow-up; gFOBT = guaiac-based fecal occult blood test; Hemoccult II S = Hemoccult II SENSA; HMO = health maintenance organization; mo = months; nat = national; NSP = national screening program; sms = text message; org = organization; PC = primary care; prog = program; wk = weeks; y = year; / = not available.

## Data Availability

Not applicable.

## Supplementary Materials

The following are available online at https://www.mdpi.com/2072-6694/13/7/1520/s1. Supplementary Materials. Online database search strategy, PubMed (legacy version); Table S1. Risk of bias assessment of randomized and cluster-randomized studies included in systematic review (Tool:RoB 2); Table S2. Invitation characteristics and gFOBT/FIT usage rates among population-based randomized and cluster-randomized studies performed within a national screening program; Table S3. Invitation characteristics and gFOBT/FIT usage rates among other population-based randomized studies; Table S4. Invitation characteristics and gFOBT/FIT usage rates among primary care-based randomized studies; Table S5. Invitation characteristics and gFOBT/FIT usage rates among randomized and cluster-randomized studies from safety net, FQHC, and Medicaid settings. References [10,11,12,13,14,15,16,17,18,19,20,21,22,23,24,25,26,27,28,29,30,31,32,33,34,35,36,37,38,39,40,41,42,43] are cited in the Supplementary Materials.
